# Stage-Dependent Transmural Redistribution of NET-Associated Structures and Altered DNA Architecture in Acute Appendicitis

**DOI:** 10.3390/cells15161489

**Published:** 2026-08-19

**Authors:** Jonas Pachmann, Malik Szep, Pia Meyer zu Himmern, Max Rickes, Sara Al-Madhi, Mihailo Andric, Mirhasan Rahimli, Jessica Stockheim, Katrin Hippe, Franziska S. Karras, Ulf D. Kahlert, Roland S. Croner, Martin Herrmann, Maximilian Dölling

**Affiliations:** 1 Molecular and Experimental Surgery, Department of General, Visceral, Vascular and Transplant Surgery, Faculty of Medicine and University Hospital Magdeburg, Otto-von-Guericke University, 39120 Magdeburg, Germanymaximilian.doelling@med.ovgu.de (M.D.); 2 University Clinic for General, Visceral, Vascular and Transplantation Surgery, Faculty of Medicine, Otto-von-Guericke University, 39120 Magdeburg, Germany; 3 Institute of Pathology, Otto-von-Guericke University, 39120 Magdeburg, Germany; 4 Department of Rheumatology and Immunology and Clinical Institute of Inflammation and Immunology, Frontiers Science Center for Disease-Related Molecular Network, West China Hospital, Sichuan University, Chengdu 610041, China

**Keywords:** acute appendicitis, complicated appendicitis, neutrophil extracellular traps (NETs), citrullinated histone H3, myeloperoxidase, DNA architecture, modified DNA, cfDNA

## Abstract

**Highlights:**

**What are the main findings?**

- Acute appendicitis is associated with stage-dependent accumulation of CitH3-positive NET-associated structures that organize into large patches in advanced disease.
- NET-associated chromatin remodeling shows compartment-specific enrichment in the submucosa and muscularis and is accompanied by a shift in extracellular DNA architecture toward modified DNA relative to B-DNA.

**What are the implications of the main findings?**

- Complicated appendicitis may involve spatially organized NET-associated chromatin remodeling rather than progressive neutrophil accumulation alone.
- Circulating NET-associated and extracellular DNA markers may serve as complementary translational readouts of appendicitis-associated inflammation, but require further validation for diagnosis and severity stratification.

**Abstract:**

Neutrophil extracellular traps (NETs) contribute to antimicrobial defense but may promote tissue damage when produced in excess or insufficiently cleared. Acute appendicitis is characterized by neutrophil-driven inflammation, but NET-associated structures and their compartment-specific dynamics have not yet been systematically evaluated. We analyzed appendiceal tissue across histopathological stages of adult appendicitis patients (*n* = 60), including layer-specific assessment of citrullinated histone H3 (CitH3), myeloperoxidase (MPO), B-DNA and modified DNA. In addition, we quantified NET-associated markers in the circulation of patients with histopathologically staged appendicitis (up to *n* = 52) and controls without appendicitis (*n* = 29). CitH3- and MPO-positive deposits increased with histopathological severity, formed large patches, and showed increasing involvement of the submucosa and muscularis in advanced stages. In parallel, the DNA ratio shifted toward modified DNA conformations, while B-DNA decreased. Circulating CitH3, MPO-DNA complexes, and cfDNA increased with disease stage and distinguished acute appendicitis from non-appendicitis controls. These findings suggest that acute appendicitis is not only defined by neutrophil transmigration but also by spatial and structural remodeling of NET-associated extracellular chromatin. The shift in DNA conformational patterns most likely reflects altered extracellular chromatin processing and, consequently, differential persistence of extracellular DNA. Whether circulating NET-associated markers can support diagnosis and severity stratification of appendicitis requires further validation.

## 1. Introduction

Acute appendicitis is among the most frequent causes of acute abdominal pain and emergency surgery worldwide [1]. With increasing interest in non-operative treatment strategies for uncomplicated appendicitis, the differentiation has gained substantial clinical relevance [2]. However, clinical assessment and imaging techniques do not reliably distinguish between these entities [3], which may result in inappropriate treatment. Routinely used inflammatory markers, such as C-reactive protein (CRP) and leukocyte count reflect systemic inflammation rather than the local severity of inflammation within the appendiceal wall. Therefore, circulating biomarkers that provide information on the local inflammatory stage of acute appendicitis could add important diagnostic value. Several mechanisms, including luminal obstruction, microbiome alterations, ischemia, and immune dysregulation, have already been proposed to contribute to the pathogenesis of appendicitis [4,5,6,7], but these mechanisms have not yet translated into clinically established biomarkers for staging disease severity.

Neutrophils are the predominant circulating immune cells infiltrating the vermiform appendix during acute appendicitis [4]. To fight invading pathogens, they use multiple antimicrobial strategies, including degranulation, phagocytosis, and the release of neutrophil extracellular traps (NETs) at sites of inflammation [8,9,10]. NETs can capture and immobilize pathogens, promote their antimicrobial killing, and facilitate subsequent phagocytic clearance [11,12]. NETs consist of extracellular chromatin associated with a heterogeneous repertoire of neutrophil-derived proteins. Their molecular composition may vary depending on the activating stimulus, the local microenvironment, and the mechanism of NET formation [13]. Frequently detected NET-associated proteins include myeloperoxidase (MPO), neutrophil elastase (NE), proteinase 3 (PR3), and calprotectin [10,11,14]. NET release is associated with the citrullination of histones by PAD4 [15]. While citrullinated histone 3 (CitH3) is linked to chromatin decondensation [16], granular MPO may participate in ROS-dependent NET formation, but it is not specific for NET formation [17].

Under physiologic conditions, extracellular chromatin is effectively cleared by endogenous nucleases and phagocytes [12,18]. The torsional stress favors a structural shift from canonical B-DNA toward modified or non-B-DNA conformations [19]. The latter have been associated with increased resistance to nuclease-mediated degradation and may, consequently, promote the persistence of extracellular chromatin within inflamed tissues [20,21]. Persistent NET deposits may sustain local inflammation and contribute to proteolytic tissue damage [22,23]. Little is known about the spatial distribution and layer-specific dynamics of NETs across the appendiceal wall during acute appendicitis. Circulating NET-associated markers can be detected in peripheral blood and have been investigated as biomarkers in inflammatory diseases, including pediatric acute appendicitis [24,25]. To our knowledge, no study has integrated the spatial distribution of NETs in appendiceal tissue or circulating NET markers across histopathological stages of acute appendicitis in adults.

We postulated that dysregulated NET formation may represent a mechanism contributing to the progression from early mucosal inflammation to advanced transmural involvement. Specifically, we hypothesized that an increasing histopathological severity of acute appendicitis is characterized by (I) stage-dependent and spatially organized accumulation of NET-associated structures within the appendiceal wall, (II) changes in extracellular DNA architecture, and (III) increase in the circulating levels of NET-associated markers.

## 2. Materials and Methods

### 2.1. Study Design, Cohort, Staging and Definitions

This prospective observational study enrolled adult patients (>18 years) who were presented to the emergency department at the University Hospital Magdeburg with suspected appendicitis from December 2023 to February 2025. All participants were enrolled through the same clinical pathway for suspected acute appendicitis. Patients in whom acute appendicitis was subsequently excluded on the basis of clinical assessment, routine laboratory findings, and imaging served as the non-appendicitis serum control group. Patients with confirmed acute appendicitis underwent appendectomy and were classified as catarrhal, phlegmonous, gangrenous, or perforated/abscess appendicitis. Catarrhal and phlegmonous appendicitis were grouped as uncomplicated appendicitis, whereas gangrenous and perforated/abscess appendicitis were grouped as complicated appendicitis. Study-specific serum samples were included only after informed consent and when blood had been collected before antibiotic treatment or surgery. Patients without an eligible serum sample remained available for analyses of routine laboratory parameters and, where applicable, appendiceal tissue. Accordingly, sample sizes differed between individual analyses; exact analysis-specific sample numbers are provided in Supplementary Table S1A,B.

### 2.2. Serum Sample Collection

We collected blood (BD Vacutainer SST II Advance) prior to antibiotic or surgical intervention. Routine laboratory parameters were available for most patients as part of standard emergency care, whereas NET-associated biomarker and cfDNA analyses required separately collected study-specific serum samples. Study-specific serum samples were not available for all patients because sampling was not always feasible within the emergency care workflow. Samples were immediately stored at 4 °C and processed as soon as feasible, with a maximum interval of 4 h between blood collection and centrifugation. This interval reflected the logistical availability of study personnel, whereas routine laboratory testing, including leukocyte counts and CRP measurements, was continuously available. We centrifuged samples at 10,000× *g* for 10 min at 4 °C; serum was aliquoted and stored at −20 °C until further analysis. We minimized freeze–thaw cycles.

### 2.3. Tissue Collection

Two board-certified pathologists performed histopathological staging and assessment of appendiceal wall architecture as part of routine H&E-based diagnostics and classified appendicitis as catarrhal, phlegmonous, gangrenous, or perforated/abscess appendicitis. Biomarker results were not available to the pathology team at this time. Tissue analyses were performed using available and technically evaluable appendiceal specimens. We used non-inflamed vermiform appendix tissue from colectomy specimens (e.g., oncologic resections) without histological signs of appendiceal inflammation.

### 2.4. Immunofluorescence for Neutrophil Markers

We placed paraffin-embedded appendix sections (3–5 µm) on Fisherbrand Superfrost Plus microscope slides (22-037-246, Fisher Scientific, Pittsburgh, PA, USA), deparaffinized them (45 min at 64 °C) and cleared them in three changes of Roticlear (A538.6, Carl Roth, Karlsruhe, Germany), followed by rehydration through a graded isopropanol series (2 × 100%, 2 × 96%, 1 × 70%, all *v*/*v*; 5 min each). We performed antigen retrieval in epitope retrieval solution (10 mM sodium citrate, pH 6.0) at 90 °C for 20 min. Sections were blocked in PBS containing 1% (*w*/*v*) BSA, 2% (*v*/*v*) goat serum, 0.05% (*v*/*v*) Tween-20, 0.05% (*v*/*v*) Triton X-100, and then incubated overnight at 4 °C with primary antibodies (Supplementary Table S2). For negative controls, we incubated sections with blocking buffer instead of primary antibodies. We performed stainings as single-label immunofluorescence assays, except for the B-DNA and modified DNA experiments, which we co-stained using spectrally distinct secondary antibodies. After washing with PBS, we incubated sections with secondary antibodies for 1.5 h at room temperature in the dark. We employed Cy5-conjugated goat anti-rabbit IgG (111-175-144, Jackson ImmunoResearch, West Grove, PA, USA) and R-phycoerythrin-conjugated goat anti-mouse IgG (115-116-071, Jackson ImmunoResearch, West Grove, PA, USA) for CitH3, MPO, and modified DNA, and B-DNA, respectively (Supplementary Table S3). We stained nuclear DNA with DAPI (A4099.0010, AppliChem, Darmstadt, Germany) and propidium iodide (PI; P4641, Sigma-Aldrich, Taufkirchen, Germany), as indicated. We mounted slides in DAKO Fluorescence Mounting Medium (S3023, Agilent, Santa Clara, CA, USA) and examined them on an Aperio VERSA 8 fluorescence slide scanner (Leica Biosystems, Nussloch, Germany) with a 10× objective.

### 2.5. Qualitative Confocal NE/DNA Immunofluorescence

For complementary qualitative confocal imaging, FFPE appendix sections were deparaffinized and rehydrated, followed by heat-mediated antigen retrieval in 10 mM sodium citrate buffer (pH 6.0) at 90 °C for 20 min. After cooling to room temperature, sections were permeabilized with 0.1% (*v*/*v*) Triton X-100 in PBS for 5 min and blocked in PBS containing 5% (*v*/*v*) normal goat serum, 2% (*w*/*v*) BSA, and 0.05% (*v*/*v*) Tween-20. The sections were simultaneously incubated overnight at 4 °C with rabbit anti-neutrophil elastase (NE; ab68672, Abcam, Cambridge, UK; 1:100) and mouse monoclonal anti-DNA IgM (clone AC-30-10; CBL186, MilliporeSigma, Burlington, MA, USA; 1:100) diluted in PBS containing 1% (*w*/*v*) BSA and 0.05% (*v*/*v*) Tween-20. After washing with PBS, NE was detected using Alexa Fluor 488-conjugated donkey anti-rabbit IgG (A-21206, Thermo Fisher Scientific, Waltham, MA, USA; 1:1000), and DNA using Alexa Fluor 647-conjugated goat anti-mouse IgM (A-21238, Thermo Fisher Scientific; 1:1000). Secondary antibodies were incubated for 45 min at room temperature in the dark. Nuclear DNA was counterstained with DAPI, and sections were mounted in DAKO Fluorescence Mounting Medium (S3023, Agilent, Santa Clara, CA, USA). Confocal images were acquired using a Leica TCS SP8 confocal microscope (Leica Microsystems, Wetzlar, Germany). The images shown are single optical sections and were used for qualitative morphological assessment of the spatial association between NE-positive signal and extranuclear DNA.

### 2.6. Routine Laboratory Parameters

Total peripheral blood leukocyte counts and C-reactive protein (CRP) concentrations were obtained as part of the routine diagnostic assessment in the emergency department. CRP concentrations were determined in lithium-heparin plasma using a particle-enhanced immunoturbidimetric assay (Tina-quant C-Reactive Protein IV, CRP4) on a cobas pro c 703 clinical chemistry analyzer (Roche Diagnostics, Mannheim, Germany). Total leukocyte counts were measured in EDTA-anticoagulated whole blood using fluorescence flow cytometry on a Sysmex XR-9000 hematology analyzer (Sysmex Corporation, Kobe, Japan). Differential blood counts were not systematically obtained as part of the routine diagnostic pathway.

### 2.7. Myeloperoxidase-DNA (MPO-DNA) ELISA

We measured circulating MPO-DNA complexes in serum using a modified capture ELISA based on published protocols [26,27]. We coated 96-well Nunc MaxiSorp plates (442404, Thermo Scientific Nunc, Roskilde, Denmark) overnight at 4 °C with rabbit anti-human myeloperoxidase (07-496-I, Merck Millipore, Burlington, MA, USA, 0.5 µg/mL, 1:2000) in carbonate–bicarbonate coating buffer (0.1 M Na_2_CO_3_/0.1 M NaHCO_3_, pH 9.6; 100 µL/well). We washed the plates three times with PBS containing 0.05% (*v*/*v*) Tween-20 (PBS-T) and blocked them with 3% (*w*/*v*) BSA in PBS (200 µL/well) for 2 h at room temperature on a plate shaker. After three further washes with PBS-T, we added serum samples (40 µL/well) and incubated them for 2 h at room temperature. We then washed the plates three times with PBS-T, and added peroxidase-conjugated anti-DNA antibody (anti-DNA POD; Cell Death Detection ELISA Plus reagents, 11774425001, Roche Diagnostics, Mannheim, Germany; 1:100 in the supplied lysis buffer; 100 µL/well) for 90 min at room temperature. Plates were washed three times with PBS-T, followed by addition of TMB substrate (421101, BioLegend, San Diego, CA, USA; 50 µL/well) and incubation for 45 min in the dark. We stopped the reaction with 25% (*w*/*w*) H_2_SO_4_ (25 µL/well). We read absorbance at 450 nm (reference 620 nm) and report results as blank-subtracted mOD.

### 2.8. Citrullinated Histone H3 (CitH3) ELISA

We measured serum CitH3 using the Citrullinated Histone H3 ELISA (501620, Cayman Chemical, Ann Arbor, MI, USA) according to the manufacturer’s protocol. We incubated standards and diluted samples (100 µL/well) for 2 h at room temperature on an orbital shaker. We washed the wells five times and incubated them with HRP conjugate (100 µL/well) for 1 h. After five washes, we developed TMB (100 µL/well) for 30 min in the dark, stopped the reaction (100 µL/well), and read absorbance at 450 nm. We calculated concentrations from a blank-subtracted standard curve and report them in ng/mL.

### 2.9. Cell-Free DNA (cfDNA) Assay (PicoGreen)

We quantified circulating cell-free DNA (cfDNA) in serum as double-stranded DNA using the Quant-iT™ PicoGreen™ dsDNA Assay Kit (P7589, Thermo Fisher Scientific Inc., Waltham, MA, USA) according to manufacturer’s instructions. We diluted serum samples 1:20 in 1× TE buffer and assayed them in 96-well flat-bottom plates (655180, Greiner Bio-One, Kremsmünster, Austria). For measurement, we combined 100 µL of standards and diluted samples with 100 µL of PicoGreen working solution (1:200 in 1× TE) and incubated the plates for 5 min at room temperature protected from light. We measured fluorescence on an Infinite F200 PRO plate reader (Tecan, Männedorf, Switzerland) at 480 nm excitation and 520 nm emission. We calculated cfDNA concentrations from the standard curve (blank-subtracted) and report them in ng/mL. The PicoGreen assay detects double-stranded DNA in the cell-free serum and does not distinguish DNA conformational states or cellular origin.

### 2.10. Quantification

We performed quantitative image analyses uniformly on fluorescence images by morphometry using Photoshop CC 2018 (Adobe, Munich, Germany). For spatial tissue assessment, we operationally defined CitH3-positive extracellular structures in spatial association with extracellular DNA staining as NET-associated structures. We considered CitH3 a NET-associated histone modification, and MPO a neutrophil cytoplasmic marker. This immunofluorescence-based definition was used for spatial tissue assessment and does not establish functional NET activity. We calculated the marker burden as the product of marker-positive pixel count and mean fluorescence intensity (MFI), normalized to the corresponding tissue area. We performed whole-tissue analyses across the complete appendiceal tissue section. For layer-resolved analyses, we subdivided the appendiceal wall into mucosa, submucosa, muscularis, and serosa/subserosa. This definition was based on an initial definition of the appendix wall sections, confirmed by pathology.

### 2.11. Citrullinated Histone H3- and Myeloperoxidase-Positive Patch Analysis

We performed patch-based image analysis for CitH3- and MPO-positive signals. We defined positive signal aggregates as contiguous marker-positive regions and categorized them according to their size, expressed as pixel count. We classified patch size thresholds as small (<12 pixels), medium (12–20 pixels), or large (>20 pixels) based on visual inspection. We subsequently used large patches for layer-specific distribution and scoring analyses. We assessed layer-specific large-patch involvement using a semiquantitative four-point score: 0, no detectable signal; 1, focal or sparse signal; 2, moderate multifocal signal; and 3, extensive or confluent signal within the respective wall layer. Two investigators independently scored all samples while blinded to histopathological stage and discrepancies were resolved by consensus.

### 2.12. Statistical Analysis

We performed statistics using Stata 19 (StataCorp LLC, College Station, TX, USA). We created graphs using Prism Version 11.0.2 (GraphPad Software, Boston, MA, USA). We assessed differences across histopathological groups using the Kruskal–Wallis test and evaluated ordered stage-dependent trends using the Cuzick trend test. For DNA architecture tissue analyses, we assessed B-DNA, modified DNA, and the DNA ratio (modified DNA/B-DNA ratio) globally and within wall compartments. We based compartment-specific heatmaps of DNA architecture remodeling on Cuzick trend statistics, with colors indicating the direction and magnitude of the stage-related trend. For circulating markers, we included non-appendicitis controls as the reference category, followed by ordered appendicitis severity groups. The association between MPO-DNA complexes and CitH3 was assessed using Spearman’s rank correlation. To account for histopathological stage, a partial Spearman correlation was additionally calculated after rank transformation of both biomarkers, with histopathological stage entered as a categorical covariate. For a translational approach, we used a clinical classification in addition to the histopathological classification. In this context, we categorized appendicitis into uncomplicated (UAA) and complicated appendicitis (CAA). UAA included catarrhal (Cat) and phlegmonous (Phl), whereas CAA included gangrenous (Gan) and perforated/abscess (P/A) appendicitis. Diagnostic performance of circulating markers was assessed by receiver operating characteristic (ROC) analysis, and areas under the curve (AUCs) are reported. Differences between paired ROC curves were evaluated using DeLong’s test. Pairwise comparisons between UAA and CAA were performed using the Wilcoxon rank-sum test. We displayed strongly right-skewed circulating marker concentrations after ln(x + 1) transformation to improve graphical readability while retaining zero or near-zero values. Statistical comparisons were performed using non-parametric tests on the corresponding marker distributions. Scatterplots show individual values and medians. Displayed *p* values in figures indicate Cuzick trend test unless stated otherwise. All statistical tests were two-sided, and *p* values < 0.05 were considered statistically significant.

## 3. Results

### 3.1. CitH3-Positive Aggregate Sizes Correlate with the Histopathological Severity of Appendicitis

**Figure 1:**
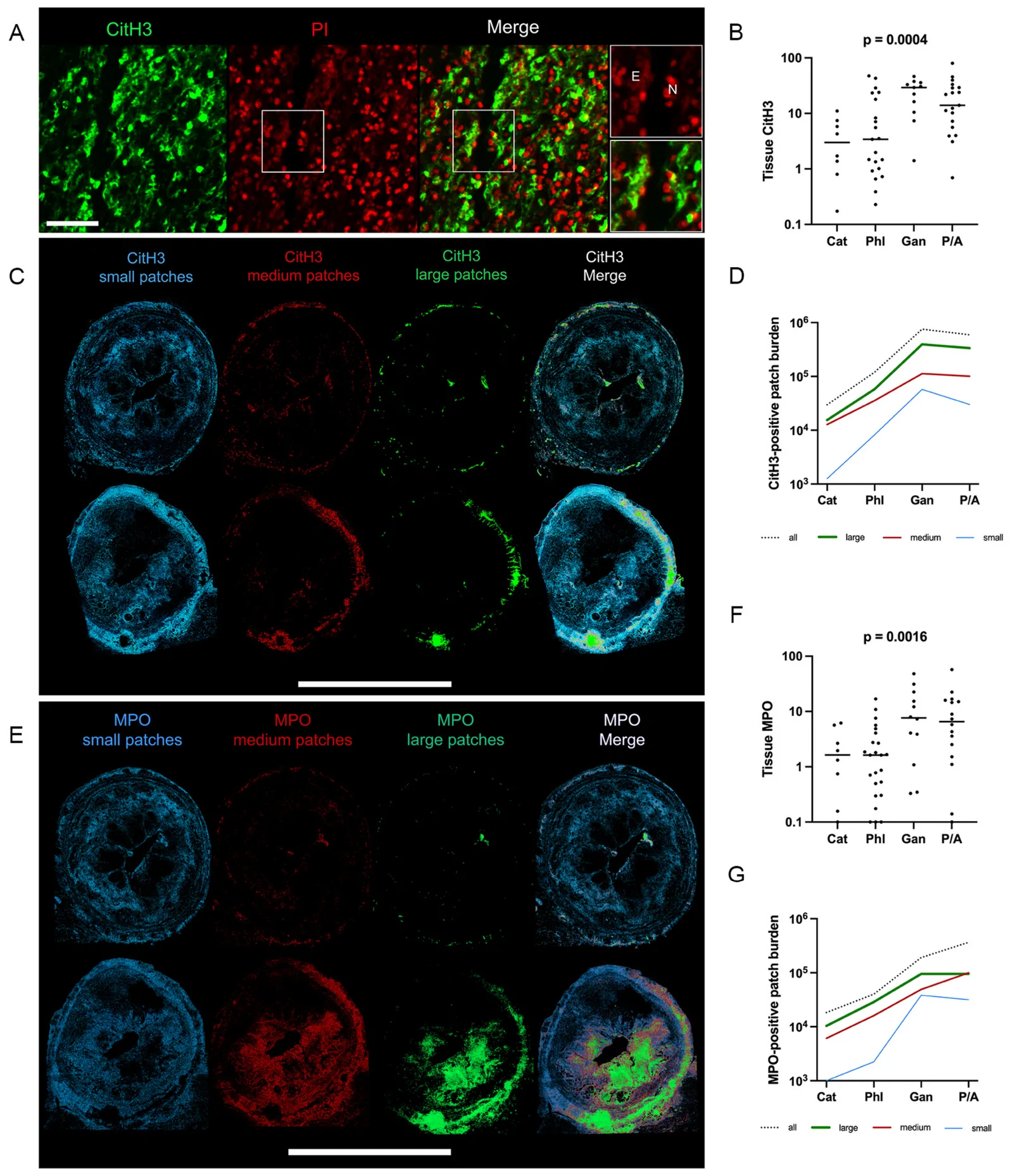
CitH3 stage-dependently forms extended larger patches in advanced appendicitis. (**A**) Representative immunofluorescence image demonstrates CitH3 (green) in spatial association with extracellular DNA (propidium iodide; red) in inflamed appendiceal tissue. Insets show magnified views of the boxed regions and highlight nuclear [N] and extracellular DNA [E]. Scale bar: 100 µm. (**B**) Quantification of CitH3 across catarrhal (Cat, *n* = 9), phlegmonous (Phl, *n* = 21), gangrenous (Gan, *n* = 11), and perforated/abscess appendicitis (P/A, *n* = 19). (**C**) Representative images of entire wall of vermiform appendix illustrating size-based segmentation of CitH3-positive patches in catarrhal (Cat) and gangrenous (Gan) appendicitis. Scale bar: 1 cm. (**D**) Quantification of CitH3 patches stratified by patch size. (**E**) Representative images of entire wall of vermiform appendix illustrating size-based segmentation of MPO-positive patches in catarrhal (Cat) and gangrenous (Gan) appendicitis. Scale bar: 1 cm. (**F**) Quantification of MPO across catarrhal (Cat, *n* = 9), phlegmonous (Phl, *n* = 21), gangrenous (Gan, *n* = 11), and perforated/abscess appendicitis (P/A, *n* = 19). (**G**) Quantification of MPO patches stratified by patch size.

Routine H&E histology confirmed the stage-dependent inflammatory involvement of the appendiceal wall. Representative phlegmonous and gangrenous specimens illustrate the distribution of acute inflammatory infiltrates (Supplementary Figure S1). Immunofluorescence analysis of inflamed appendiceal tissue (*n* = 60) identified CitH3-positive extracellular structures in spatial association with extracellular DNA, which were operationally classified as NET-associated structures (Figure 1A). Representative confocal imaging additionally showed NE-positive signal in close spatial association with extranuclear DNA (Supplementary Figure S2). Based on these findings, we quantified whole-tissue burden of the NET-associated histone modification CitH3 and the neutrophil granule enzyme myeloperoxidase (MPO). CitH3-positive tissue burden increased significantly across histopathological stages of appendicitis (*p* = 0.0004), with the highest values observed in gangrenous and perforated/abscess appendicitis (Figure 1B). Non-inflamed tissue controls (*n* = 3) and catarrhal appendicitis did not differ in CitH3 burden (*p* = 0.7815). Subsequent analyses were therefore restricted to appendicitis cases. Analogous to CitH3, MPO was increased across histopathological stages with the highest in gangrenous and perforated/abscess appendicitis (*p* = 0.0016; Figure 1F). Representative whole-section immunofluorescence overview of CitH3- and MPO-positive structures across appendicitis stages is shown in Supplementary Figure S3.

We assessed the spatial organization of CitH3-positive tissue areas by segmenting CitH3-positive patches and classifying them according to patch size (Figure 1C,D). While catarrhal and phlegmonous appendicitis were dominated by small- and medium-sized patches, advanced appendicitis was characterized by increased large patches (*p* = 0.0008). MPO-positive patches followed a similar pattern with increases in both total and large patch burden across the histopathological stages (Figure 1E,G). Overall, advanced appendicitis was associated with higher CitH3 and MPO tissue burden together with spatial organization into larger patches.

### 3.2. Advanced Appendicitis Is Marked by High-Grade CitH3-Positive Patches in Submucosa and Muscularis

**Figure 2:**
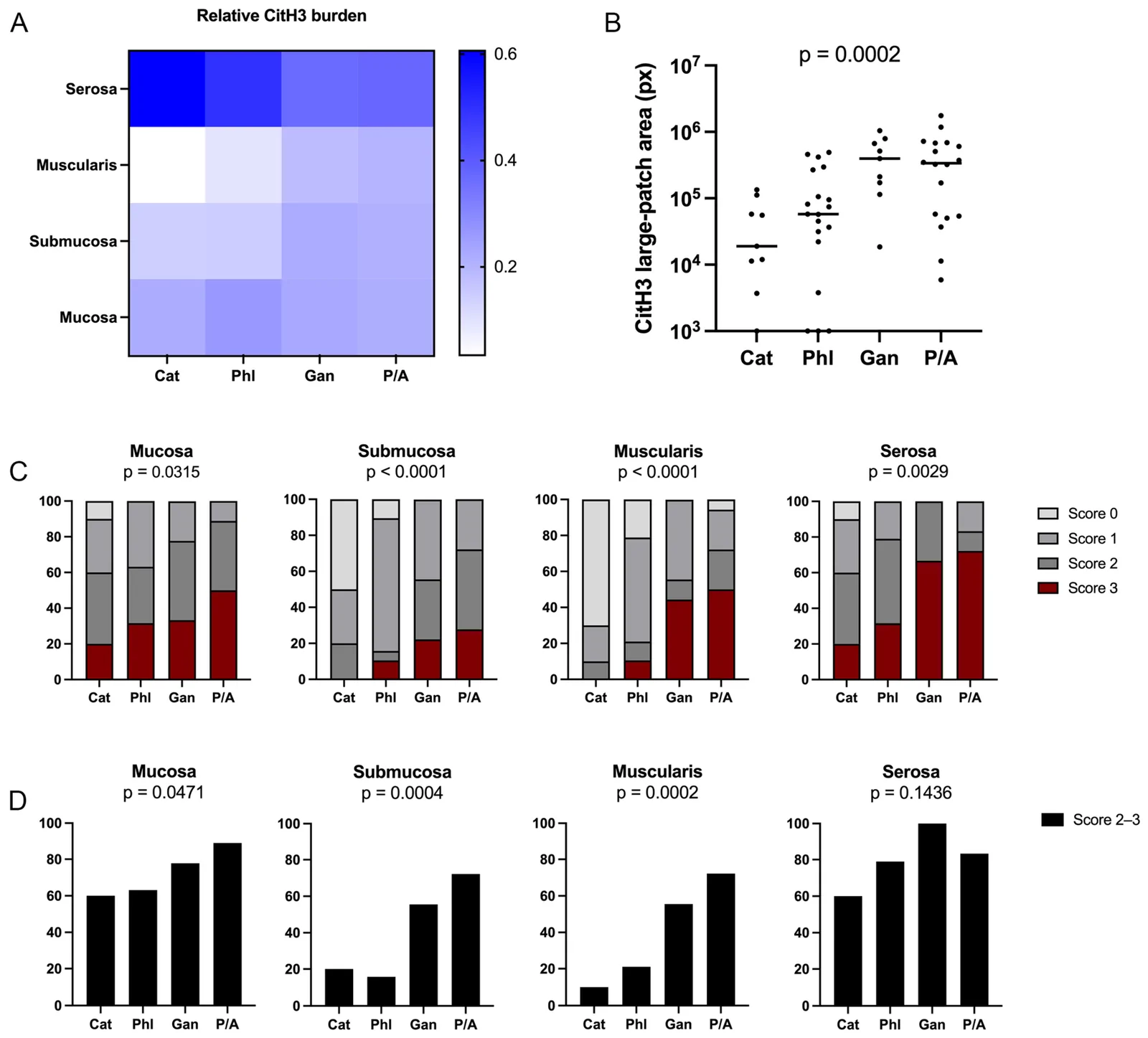
CitH3-positive aggregates emerge in submucosa and muscularis and form high-grade patches increasing with histopathological severity. (**A**) The heatmap reveals the relative distribution of CitH3 burden across appendiceal wall compartments. Color intensity represents the fraction of total CitH3-positive burden within each histopathological category. (**B**) Quantification of total CitH3-positive large-patch area across catarrhal (Cat, *n* = 9), phlegmonous (Phl, *n* = 19), gangrenous (Gan, *n* = 9), and perforated/abscess appendicitis (P/A, *n* = 18). (**C**) Layer specific distribution of CitH3-positive large-patch scores across appendiceal wall compartments (score 0–3). (**D**) Percentage of patients with high CitH3 large-patch scores (score 2–3) within each compartment.

We next investigated whether NET-associated structures showed layer-specific redistribution across the appendiceal wall. Representative annotations of appendiceal wall compartments are shown in Supplementary Figure S4. Layer-normalized CitH3 burden demonstrated a compartment-specific redistribution across histopathological categories (Figure 2A). While the relative CitH3 contribution was highest in the serosal compartment in catarrhal and phlegmonous appendicitis, advanced appendicitis presents increasing relative involvement of deeper wall compartments, particularly the submucosa and muscularis. Consistent with the increasing abundance of large CitH3-positive patches, total large-patch area increased across histopathological stages (Kruskal–Wallis *p* = 0.0008; Cuzick *p* = 0.0002; Figure 2B). MPO-based analyses resulted in a similar but less pronounced pattern (Supplementary Figure S5).

To classify the layer-specific large-patch involvement, we assessed a semiquantitative score ascending from 0 (no signal) to 3 (extensive or confluent signal). The scoring of CitH3-positive large patches showed a transmural redistribution toward deeper wall layers, with the most pronounced changes in submucosa and muscularis (Figure 2C). High-level scores (score 2–3) increased most prominently in these compartments, whereas the serosal compartment did not show a significant ordered trend (Figure 2D).

### 3.3. Extracellular B-DNA Loss Reveals Compartment-Specific DNA Remodeling in Advanced Appendicitis

**Figure 3:**
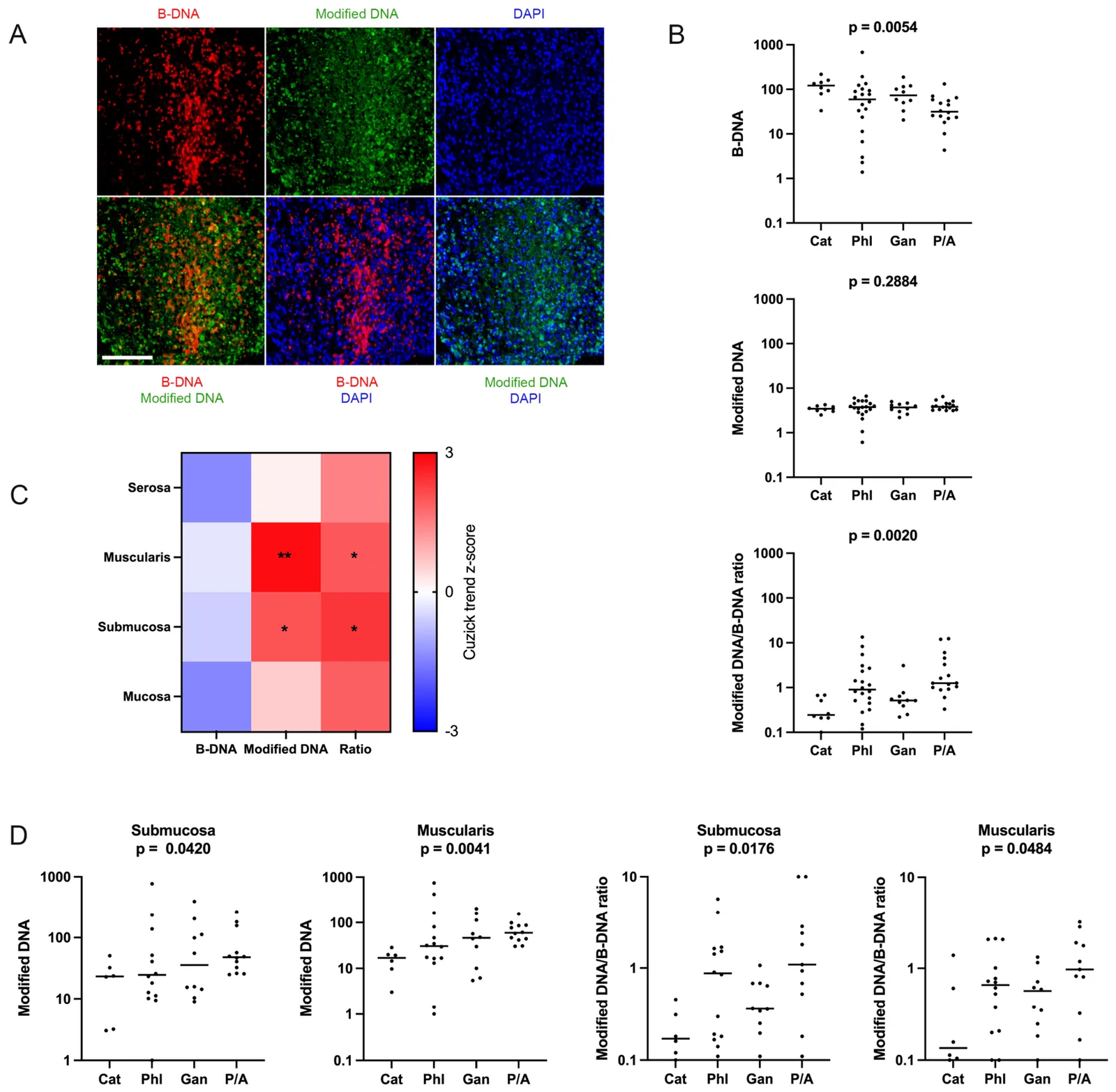
Loss of extracellular B-DNA shifts DNA toward modified DNA in compartment-specific patterns. (**A**) Immunofluorescence reveals extracellular B-DNA (red), modified DNA (green), DAPI (blue), and merged images in inflamed appendiceal tissue. Scale bar: 100 µm. (**B**) Quantification of total B-DNA, total modified DNA, and the modified DNA/B-DNA ratio across catarrhal (Cat, *n* = 8), phlegmonous (Phl, *n* = 20), gangrenous (Gan, *n* = 10), and perforated/abscess appendicitis (P/A, *n* = 16). (**C**) Compartment-resolved heatmap summarizing Cuzick trend z-scores for B-DNA, modified DNA, and the modified DNA/B-DNA ratio across appendiceal wall layers. Blue indicates decreasing and red indicates increasing values across ordered histopathological stages. Asterisks indicate significant Cuzick trend tests (* *p* < 0.05, ** *p* < 0.01). (**D**) Selected compartment-specific plots showing modified DNA burden and the modified DNA/B-DNA ratio in the submucosa and muscularis across histopathological stages.

We next focused on extracellular DNA architecture patterns across histopathological stages. Representative immunofluorescence images demonstrated extracellular B-DNA and modified DNA in inflamed appendiceal tissue (Figure 3A). Quantitative analysis revealed a decrease in total B-DNA burden (Kruskal–Wallis *p* = 0.0074; Cuzick trend *p* = 0.0054; Figure 3B). Total modified DNA burden did not differ between stages, whereas the DNA ratio (Modified DNA/B-DNA ratio) increased with histopathological severity (Kruskal–Wallis *p* = 0.0004; Cuzick trend *p* = 0.0020).

Compartment-specific analyses showed that DNA remodeling was not uniformly distributed across the appendiceal wall (Figure 3C). The most prominent changes were observed in the submucosa and muscularis (Supplementary Table S4). In the submucosa, DNA ratio increased with disease severity (Kruskal–Wallis *p* = 0.0252; Cuzick trend *p* = 0.0176) and was accompanied by a stage-related increase in modified DNA burden (Cuzick trend *p* = 0.0420; Figure 3D). In the muscularis, modified DNA burden differed between histopathological stages and showed a strong ordered increase (Kruskal–Wallis *p* = 0.0298; Cuzick trend *p* = 0.0041), while the DNA ratio exhibited a weaker but ordered trend.

### 3.4. Circulating Inflammatory and NET-Associated Markers Increase with Histopathological Severity

**Figure 4:**
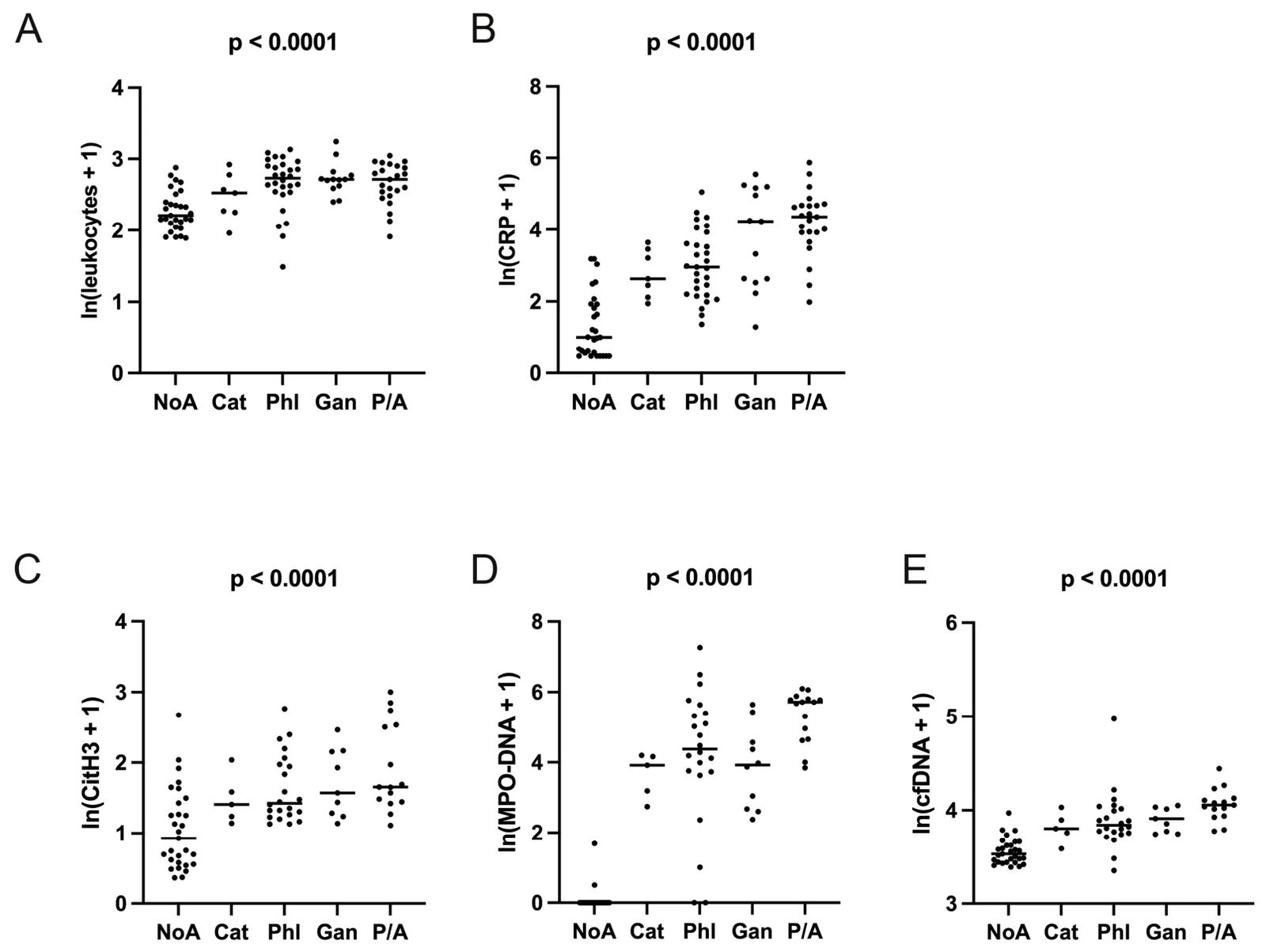
Circulating NET-associated markers increases from no appendicitis toward advanced appendicitis. (**A**,**B**) Circulating leukocytes and CRP as routine inflammatory markers in patients with no appendicitis (NoA) and across catarrhal (Cat), phlegmonous (Phl), gangrenous (Gan), and perforated/abscess appendicitis (P/A). Sample sizes were: leukocytes, *n* = 7/28/13/23; CRP, *n* = 7/29/13/23 for Cat/Phl/Gan/P/A, respectively. (**C**–**E**) CitH3 and MPO-DNA complexes served as NET-associated markers, and cfDNA as a circulating extracellular DNA marker. Sample sizes were: CitH3, *n* = 5/22/9/15; MPO-DNA, *n* = 5/22/10/15; and cfDNA, *n* = 5/22/9/15. NoA was *n* = 29 in all markers. Data are shown as ln(x + 1)-transformed values. Reported *p* values were calculated using Cuzick’s non-parametric test for trend across the five ordered diagnostic groups.

After characterizing tissue inflammation, we assessed the systemic immune response. Circulating leukocyte counts and CRP levels increased across ordered categories from no appendicitis (NoA) to perforated/abscess appendicitis (P/A) (Kruskal–Wallis *p* < 0.0001; Cuzick trend *p* < 0.0001 for both markers; Figure 4A,B). In parallel, CitH3 and cfDNA levels differed between categories and showed a positive ordered trend (Kruskal–Wallis *p* = 0.0001; Cuzick trend *p* < 0.0001; Figure 4C,E). MPO-DNA complexes were largely absent in no appendicitis and increased across ordered appendicitis stages (Kruskal–Wallis *p* = 0.0001; Cuzick trend *p* < 0.0001; Figure 4D). Across the entire serum cohort, MPO-DNA complexes and CitH3 were positively correlated in the unadjusted analysis (Spearman’s ρ = 0.488, *p* < 0.001; *n* = 80). After adjustment for histopathological stage, the association was no longer significant (partial Spearman’s ρ = 0.069, *p* = 0.552; *n* = 80).

### 3.5. Circulating NET-Associated Markers Identify Appendicitis While cfDNA Emerges as the Strongest Circulating Extracellular DNA-Associated Marker of Complicated Appendicitis

**Figure 5:**
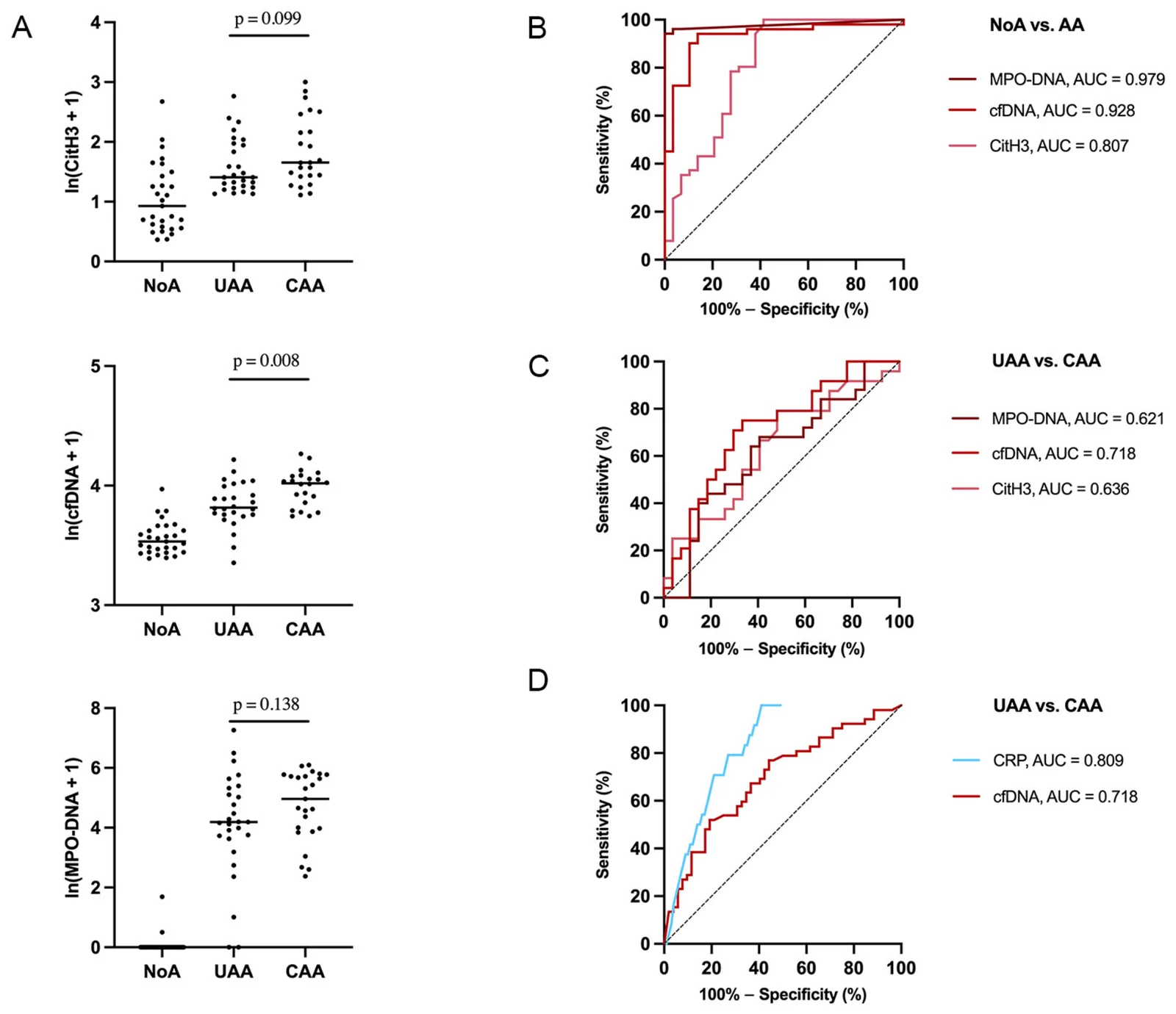
cfDNA best discriminated against complicated disease among NET-associated and extracellular DNA markers. (**A**) Circulating MPO-DNA, cfDNA, and CitH3 levels across no appendicitis (NoA; *n* = 29), uncomplicated (UAA; Cat or Phl; *n* = 27) and complicated (CAA; Gan or P/A; *n* = 24 for CitH3 and cfDNA; MPO-DNA *n* = 25) appendicitis. Data are shown as ln(x + 1)-transformed values. The *p*-values indicate Wilcoxon rank-sum tests comparing UAA with CAA. (**B**) ROC analysis for discrimination of (NoA, *n* = 29) from acute appendicitis (AA, *n* = 51 for CitH3 and cfDNA; MPO-DNA *n* = 52) using circulating MPO-DNA complexes, cfDNA, and CitH3. (**C**) ROC analysis for discrimination of UAA from CAA using circulating MPO-DNA complexes (*n* = 27/25), cfDNA (*n* = 27/24), and CitH3 (*n* = 27/24). (**D**) ROC analysis comparing CRP and cfDNA for discrimination of UAA from CAA in the paired subset of patients with both markers. CRP did not differ from cfDNA (AUC 0.809 vs. 0.718; *p* = 0.2633 applying DeLong’s test).

To determine whether circulating NET-associated and extracellular DNA markers could support clinically oriented classification of appendicitis, histopathological categories were grouped into no appendicitis (NoA), uncomplicated appendicitis (UAA; catarrhal and phlegmonous appendicitis), and complicated appendicitis (CAA; gangrenous and perforated/abscess appendicitis). We first compared marker levels across the clinically defined groups NoA, UAA, and CAA (Figure 5A). In pairwise UAA versus CAA comparisons, cfDNA was significantly higher in CAA (*p* = 0.008), whereas CitH3 and MPO-DNA showed non-significant trends toward higher values in CAA (*p* = 0.099 and *p* = 0.138). ROC analysis demonstrated that circulating NET-associated and extracellular DNA markers strongly discriminated NoA from acute appendicitis (AA). MPO-DNA complexes showed the highest diagnostic performance for NoA versus AA (AUC = 0.979), followed by circulating cfDNA (AUC = 0.928) and CitH3 (AUC = 0.807; Figure 5B). ROC analysis for UAA versus CAA showed only moderate discrimination. cfDNA provided the strongest discrimination between UAA and CAA (AUC = 0.718), whereas CitH3 (AUC = 0.636) and MPO-DNA complexes (AUC = 0.621) showed weaker performance (Figure 5C; Supplementary Table S5). CRP and cfDNA showed comparable results for distinguishing UAA from CAA (AUC = 0.809 vs. 0.718), without significant difference (DeLong’s test, *p* = 0.2633; Figure 5D). Exploratory multivariable ROC models combining CRP with cfDNA, with or without leukocyte count, showed minor numerical improvement over CRP alone and did not significantly improve discrimination of CAA (Supplementary Figure S6).

## 4. Discussion

Several mechanisms have been proposed for the pathogenesis of appendicitis, but neutrophil infiltration of the appendiceal wall remains a defining histopathological feature [4]. Our findings indicate that acute appendicitis is not merely a progressive neutrophil-dominated inflammatory response but rather involves spatially organized NET-associated tissue remodeling.

NETs are released upon neutrophil activation by various stimuli, such as microbial stimuli, pathogen-associated molecular patterns, damage-associated molecular patterns, and cytokines [28,29]. As assumed in neutrophil-driven inflammation, we found a substantial increase in the neutrophil-associated marker MPO in appendiceal tissue. The parallel increase in MPO and CitH3 across histopathological stages is consistent with progressive neutrophil-rich inflammation and suggests that NET-associated mechanisms contribute to the local inflammatory tissue response in acute appendicitis. We observed the organization of CitH3 into larger patches in advanced stages of appendicitis which may reflect focal inflammatory hotspots with local accumulation and persistence of NET-associated chromatin, rather than a homogeneous increase in neutrophil-derived markers. Previous studies have described that persisting NETs may sustain local inflammation and contribute to tissue damage through the retention of matrix-degrading enzymes, including matrix metalloproteinase-9 and the serine proteases neutrophil elastase and proteinase 3 [23,30,31].

Damage-associated molecular patterns (DAMPs) are endogenous danger molecules produced in the context of cellular damage or stress [32]. NET-associated proteolytic activity has the potency to damage and weaken the extracellular matrix and expose matrix-derived DAMPs, by which persistent NET-associated enzymes may shift local inflammation toward destructive transmural involvement [33].

NET-associated tissue patterns were associated with increasing histopathological severity and reflected the extent of appendiceal wall involvement in advanced appendicitis. Across inflammatory stages, the extent of appendiceal wall involvement varied markedly, with prominent accumulation of NET-associated signals in the submucosa and muscularis during advanced appendicitis. This is biologically plausible because the submucosa is a connective tissue- and lymphoid tissue-rich compartment that may facilitate immune cell recruitment and edema formation [34]. The muscularis represents a structurally critical layer for appendiceal wall integrity [4]. For this reason, the submucosa and the muscularis could serve as critical transition zones where local tissue remodeling progresses toward destructive appendicitis.

The analysis of tissue DNA architecture added another non-canonical NET-associated marker. Extracellular DNA generated by NET formation in appendiceal tissue is not static and may adopt modified or non-B-DNA conformations under inflammatory stress. Our observation of the decreasing B-DNA burden and simultaneous rising DNA ratio suggests a relative shift toward a modified DNA-based scaffold. Physiologically, extracellular B-DNA and NET-derived chromatin are degraded by endogenous nucleases such as DNase1 and cleared by macrophages [12,18]. Delayed clearance may allow extracellular chromatin to persist as a matrix that retains cytotoxic histones, proteases, and DAMP-associated immunostimulatory activity [35]. Based on this concept, the observed shift toward modified DNA relative to B-DNA may reflect altered extracellular chromatin processing and differential persistence rather than the simple accumulation of extracellular DNA [20,21]. This interpretation is supported by recent observations, where granulomas contained Z-form DNA conformations, indicating that extracellular DNA architecture can be altered and persist within inflamed tissue contexts [36]. The DNA-remodeling in our appendix study tended to be compartment-specific analogous to CitH3, again among the key wall layers, submucosa, and muscularis. This spatial overlap suggests that NET formation provides an important source of extracellular chromatin, but the resulting DNA architecture also depends on local nuclease activity.

Acute appendicitis is clinically classified into uncomplicated (UAA) and complicated acute appendicitis (CAA) to guide physicians in their therapeutic decisions. While appendectomy is still a common therapy for appendicitis after diagnosis, selected cases of patients with UAA might be treated with non-operative therapy [37,38]. Reliable identification of CAA remains challenging because routine systemic inflammatory markers, such as CRP and leukocyte count, are limited in distinguishing UAA from CAA across different clinical classification systems [39], and do not directly report the extent of transmural inflammation. In line with this, previous EAES-based analyses demonstrated that CRP increases with disease severity and provides moderate discrimination of CAA, whereas leukocyte count performs less reliably [40,41]. We therefore assessed whether circulating NET-markers and cfDNA reflect histopathological severity and clinically oriented appendicitis categories. We observed that NET-markers increased with histopathological disease stage and strongly distinguished patients without appendicitis from those with acute appendicitis. Thus, the local tissue alterations were accompanied by a systemic NET-associated biomarker signature. MPO-DNA complexes and CitH3 were moderately correlated across the entire serum cohort; however, this association was no longer statistically significant after adjustment for histopathological stage. The circulating markers are not specific to the appendix and may reflect systemic neutrophil activation or extracellular chromatin release originating from other tissues. They should therefore not be interpreted as direct measures of appendiceal NET formation. cfDNA best discriminated between UAA and CAA out of NET-associated and extracellular DNA markers and showed comparable discrimination to CRP between UAA and CAA. cfDNA likely captures cell death, tissue damage, and extracellular DNA [42]. For this reason, it should be interpreted as a marker of circulating extracellular DNA burden rather than a direct surrogate of NET formation [43].

Exploratory multivariable models combining CRP and leukocytes with cfDNA produced only minor improvements and did not significantly improve discrimination compared with CRP alone. These findings suggest that cfDNA may capture biological information complementary to standard inflammatory markers but must not be considered equivalent or as a replacement.

Our findings are consistent with previous pediatric studies demonstrating the diagnostic value of markers for circulating NETs where serum cfDNA and CitH3 predict appendicitis in children and that CitH3 was able to distinguish UAA from CAA [25]. In our adult cohort, however, CitH3 showed weaker diagnostic value in serum than cfDNA or MPO-DNA complexes, despite being a strong tissue-associated marker of NET-associated histone modification. A possible explanation is that serum levels of NET-markers depend on many factors, such as vascular involvement, DNA conformation, phagocytosis, and disease timing. We conclude that these markers represent complementary biological dimensions of CAA.

This was a single-center cohort, and sample availability differed between sera and tissues. Due to the complexity of NET-associated inflammation, this cross-sectional study has several limitations. Because NET-associated structures were identified by immunofluorescence and confocal imaging, this approach cannot fully define functional NETs or distinguish all possible sources of extracellular chromatin. Also, these parallel findings alone do not establish that every CitH3-positive extracellular structure is derived exclusively from neutrophils. As mentioned above, no direct DNase assays were performed, which limits conclusions regarding NET clearance or DNA persistence. Because differential blood counts were not systematically obtained as part of the routine emergency diagnostic pathway, absolute neutrophil counts were unavailable for a substantial proportion of the cohort. Accordingly, total leukocyte counts cannot be regarded as a surrogate for circulating neutrophil numbers. A further limitation of this study was the interval of up to 4 h between blood collection and centrifugation. Although all samples were processed according to the same predefined protocol, ex vivo neutrophil activation and release of NET-associated components during this interval cannot be excluded. Therefore, the observed biomarker patterns should primarily be interpreted as exploratory relative differences between clinical groups rather than as definitive absolute concentrations. Moreover, MPO is neither NET-specific nor appendix-specific and reflects neutrophil-rich inflammation more broadly than CitH3. Furthermore, direct markers of MPO-mediated oxidative damage, including 4-hydroxynonenal, malondialdehyde, and 3-chlorotyrosine, as well as markers of extracellular matrix remodeling such as matrix metalloproteinases, were not assessed. Their inclusion in future studies may help clarify the relationship between neutrophil activation, oxidative tissue injury, and disease progression.

## Data Availability

The data presented in this study are available from the corresponding author upon reasonable request and subject to institutional, legal, and ethical restrictions related to human tissue and patient-derived clinical information.

## Supplementary Materials

The following supporting information can be downloaded at: https://www.mdpi.com/article/10.3390/cells15161489/s1; Figure S1: Representative architectural histology of phlegmonous and gangrenous acute appendicitis; Figure S2: Representative confocal imaging of neutrophil elastase (NE) and DNA in acute appendicitis; Figure S3: Whole-section immunofluorescence overview of CitH3- and MPO-positive structures across appendicitis severity; Figure S4: Representative annotation of appendiceal wall compartments for layer-resolved quantification; Figure S5: MPO-positive large-patch analysis supports a less pronounced compartment-specific remodeling pattern; Figure S6: Exploratory multivariable ROC models for discrimination of complicated appendicitis; Table S1A: Patient and cohort characteristics; Table S1B: Analysis-specific sample availability; Table S2: Primary antibodies; Table S3: Secondary antibodies; Table S4: Compartment-specific Cuzick trend statistics for DNA architecture markers and CitH3; Table S5: ROC performance and Wilcoxon group comparisons for UAA versus CAA.

## Abbreviations

The following abbreviations are used in this manuscript:

NoA	no appendicitis
AA	acute appendicitis
UAA	uncomplicated appendicitis
CAA	complicated appendicitis
NETs	neutrophil extracellular traps
CitH3	citrullinated histone H3
NE	neutrophil elastase
MPO	myeloperoxidase
ROS	reactive oxygen species
cfDNA	cell-free DNA
DAMPs	damage-associated molecular patterns
