# Supplementary material for: Stage-Dependent Transmural Redistribution of NET-Associated Structures and Altered DNA Architecture in Acute Appendicitis
Source: Cells. 2026 Aug 19;15(16):1489. doi: 10.3390/cells15161489 (PMC13511748; doi:10.3390/cells15161489)
Supplement: Supplementary file 1 [file cells-15-01489-s001.zip › cells-4461576-supplementary.pdf]

SUPPLEMENTS

Supplementary Table S1 A: Patient and cohort characteristics

**Parameters**		**n, n (%) or median (IQR)**
Sex (overall cohort, n = 107)	Male	61 (57.0)
	Female	46 (43.0)
Age, years (overall cohort, n = 107)		42 (30–61)
**No appendicitis (NoA);** n = 32		32
**Uncomplicated (UAA);** n = 39	Catarrhal	10
	Phlegmonous	29
**Complicated (CAA);** n = 36	Gangrenous	13
	Perforated/Abscess	23

Supplementary Table S1 B: Analysis-specific sample availability

Analysis	NoA/Non-inflamed Tissue Controls	Catarrhal	Phlegmonous	Gangrenous	P/A	Total analyzed
Enrolled clinical cohort	32	10	29	13	23	107
Leukocyte count	29	7	28	13	23	100
CRP	29	7	29	13	23	101
Serum CitH3	29	5	22	9	15	80
Serum MPO–DNA	29	5	22	10	15	81
Serum cfDNA	29	5	22	9	15	80
Appendiceal tissue	3	9	21	11	19	63

UAA comprised catarrhal and phlegmonous appendicitis; CAA comprised gangrenous and perforated/abscess appendicitis. The clinical NoA group was defined on the basis of clinical assessment, routine laboratory findings, and imaging. Patients without an eligible serum sample remained available for routine laboratory and tissue-based analyses, where applicable. Appendicitis stages were assigned according to routine histopathological assessment of the resected appendix. For routine laboratory and serum analyses, this column refers to the clinical NoA group; for appendiceal tissue analysis, it refers to non-inflamed appendix controls obtained from colectomy specimens.

Supplementary Table S2: Primary antibodies

**Primary antibodies**	**Host/Isotype**	**Supplier (catalog no.)**	**Dilution**
Citrullinated Histone H3 (CitH3)	Rabbit IgG	Abcam (ab5103)	1:300
Myeloperoxidase (MPO)	Rabbit IgG	Abcam (ab9535)	1:200
B-DNA (dsDNA)	Mouse IgG2a, kappa	Abcam (ab27156)	1:50
Z-DNA (clone Z22)	Rabbit IgG, kappa	Absolute Antibody (Ab00783-23.0)	1:100

Primary antibodies were prepared at the indicated dilution in blocking buffer. Signals detected by Z-DNA antibody are referred to as modified DNA. B-DNA and modified DNA were co-stained, whereas all other targets were stained as single-label assays.

Supplementary Table S3: Secondary antibodies

Secondary antibodies	Host/Isotype	Supplier (catalog no.)	Dilution
Cy5-conjugated AffiniPure™	goat anti-rabbit IgG (H+L)	Jackson (111-175-144)	1:400
R-phycoerythrin-conjugated AffiniPure™	goat anti-mouse IgG (H+L)	Jackson (115-116-071)	1:400

Secondary antibodies were diluted in blocking buffer and incubated for 1.5 h at room temperature in the dark. Cy5-conjugated goat anti-rabbit IgG was used for rabbit primary antibodies, and R-phycoerythrin-conjugated goat anti-mouse IgG was used for mouse primary antibodies.

Supplementary Table S4: Compartment-specific Cuzick trend statistics for DNA architecture markers and CitH3

Compartment	Marker	Cuzick z	p value
**Total**	B-DNA	−2.785	0.0054
**Total**	Modified DNA	1.062	0.2884
**Total**	Modified DNA/B-DNA index	3.095	0.0020
**Mucosa**	B-DNA	−1.435	0.1512
**Mucosa**	Modified DNA	0.596	0.5513
**Mucosa**	Modified DNA/B-DNA index	1.896	0.0579
**Mucosa**	CitH3	3.443	0.0006
**Submucosa**	B-DNA	−0.582	0.5605
**Submucosa**	Modified DNA	2.034	0.0420
**Submucosa**	Modified DNA/B-DNA index	2.374	0.0176
**Submucosa**	CitH3	3.555	0.0004
**Muscularis**	B-DNA	−0.316	0.7517
**Muscularis**	Modified DNA	2.869	0.0041
**Muscularis**	Modified DNA/B-DNA index	1.974	0.0484
**Muscularis**	CitH3	4.479	<0.0001
**Serosa**	B-DNA	−1.405	0.1601
**Serosa**	Modified DNA	0.169	0.8654
**Serosa**	Modified DNA/B-DNA index	1.481	0.1387
**Serosa**	CitH3	3.212	0.0013

Supplementary Table S5: ROC performance and Wilcoxon group comparisons for UAA versus CAA

Marker	n UAA	n CAA	AUC	95% CI	Wilcoxon p
CitH3	27	24	0.636	0.475–0.789	0.099
MPO-DNA	27	25	0.621	0.460–0.771	0.138
cfDNA	27	24	0.718	0.565–0.849	0.008

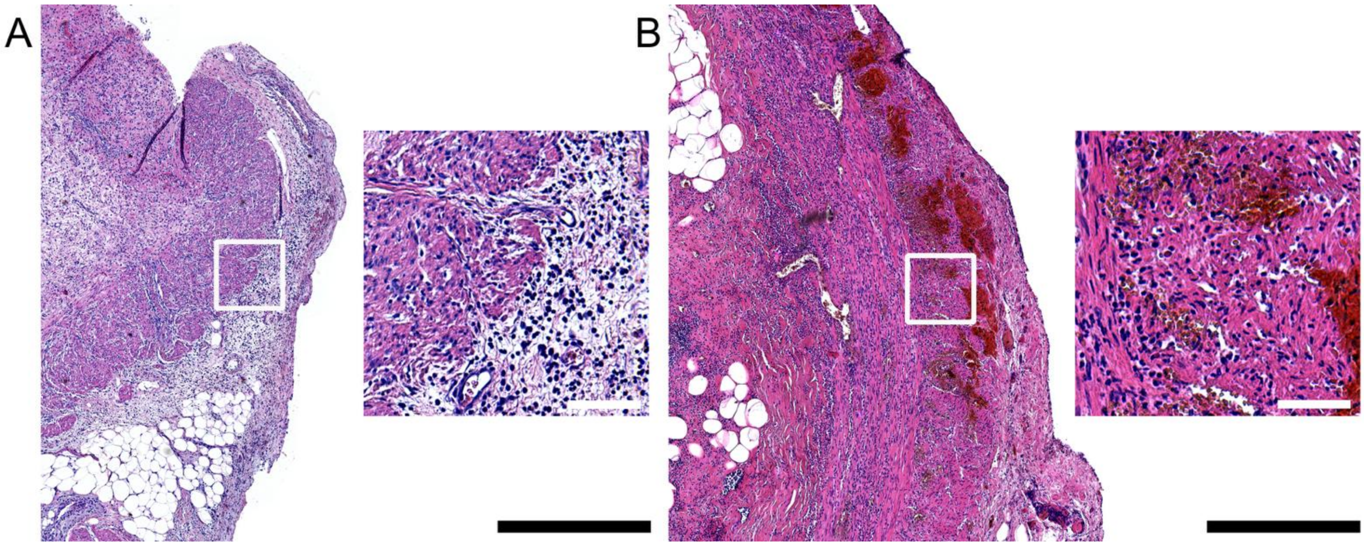

Supplementary Figure S1: Representative architectural histology of phlegmonous and gangrenous acute appendicitis

(A) Representative H&E-stained section of phlegmonous acute appendicitis demonstrating inflammatory involvement of the appendiceal wall. The boxed region is shown at higher magnification and demonstrates a dense acute inflammatory infiltrate involving the muscularis propria and adjacent subserosal tissue, including numerous cells morphologically consistent with neutrophils. (B) Representative H&E-stained section of gangrenous acute appendicitis demonstrating extensive inflammatory involvement of the appendiceal wall with associated hemorrhagic changes. The boxed region is shown at higher magnification and demonstrates dense acute inflammatory infiltration at the muscularis propria–subserosal interface, including numerous cells morphologically consistent with neutrophils. H&E, hematoxylin and eosin. Scale bars: white, 100 μm; black, 2 mm.

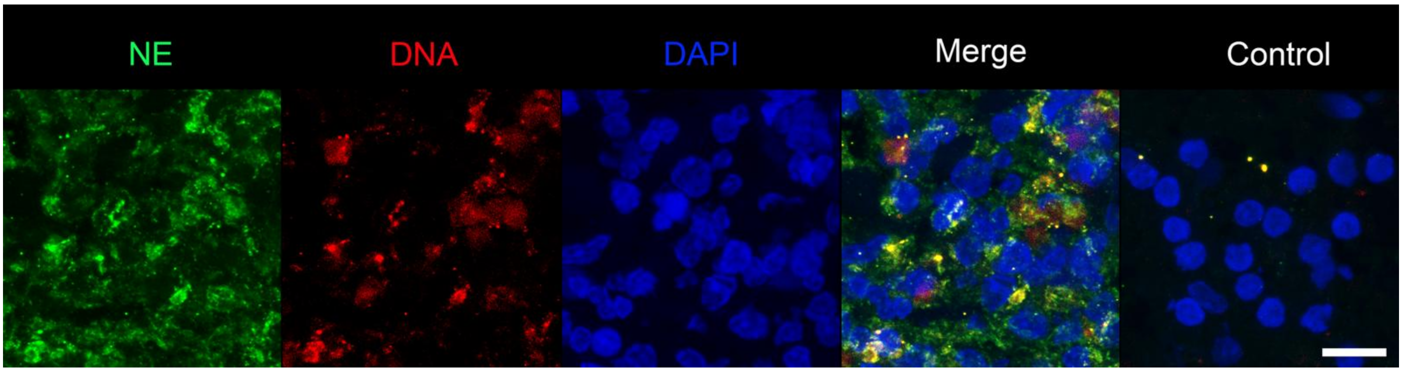

Supplementary Figure S2: Representative confocal imaging of neutrophil elastase (NE) and DNA in acute appendicitis

Representative single-plane confocal images of inflamed appendiceal tissue showing neutrophil elastase (NE, green), DNA (red), DAPI-stained nuclei (blue), and the merged channels. NE-positive signal was observed in close spatial association with extranuclear DNA signal. A corresponding control without primary antibody is shown. The image provides qualitative morphological support for neutrophil-associated extracellular DNA structures and was not used for quantitative colocalization analysis. Scale bar: 20 μm.

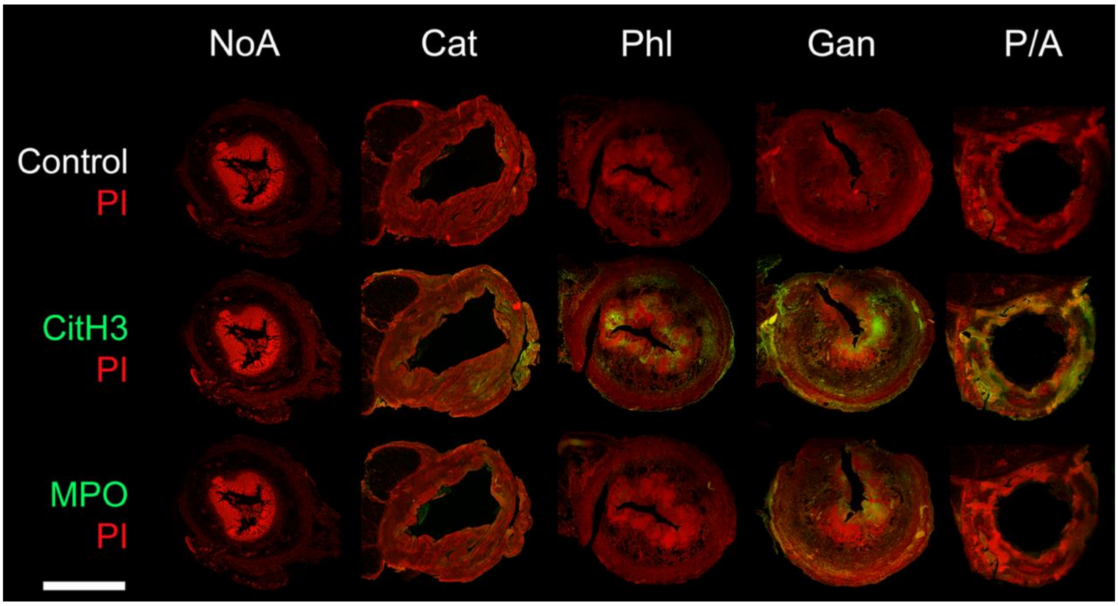

Supplementary Figure S3: Whole-section immunofluorescence overview of CitH3- and MPO-positive structures across appendicitis severity

Representative whole-section images of non-inflamed appendix tissue (NoA) and catarrhal (Cat), phlegmonous (Phl), gangrenous (Gan), and perforated/abscess (P/A) appendicitis are shown. Control panels represent no-primary-antibody controls with PI counterstaining. CitH3/PI and MPO/PI stainings illustrate the stage-associated accumulation and spatial expansion of NET-associated CitH3-positive and neutrophil-associated MPO-positive structures. P/A appendicitis displayed marked morphological heterogeneity. Scale bar: 5 mm.

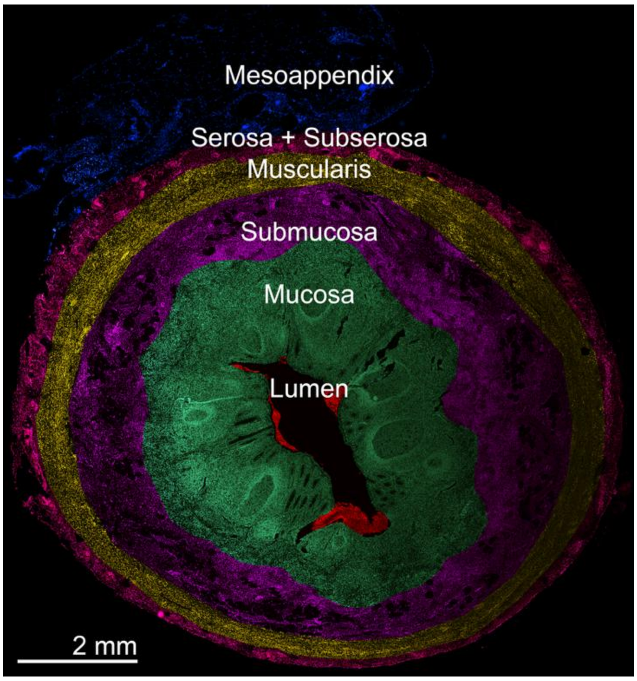

Supplementary Figure S4: Representative annotation of appendiceal wall compartments for layer-resolved quantification

Representative whole-section appendix image illustrating the anatomical regions used for compartment-specific image analysis. The appendiceal wall was subdivided into mucosa, submucosa, muscularis, and serosa/subserosa. Lumen and mesoappendix were excluded from quantitative wall-layer analyses. Scale bar: 2 mm.

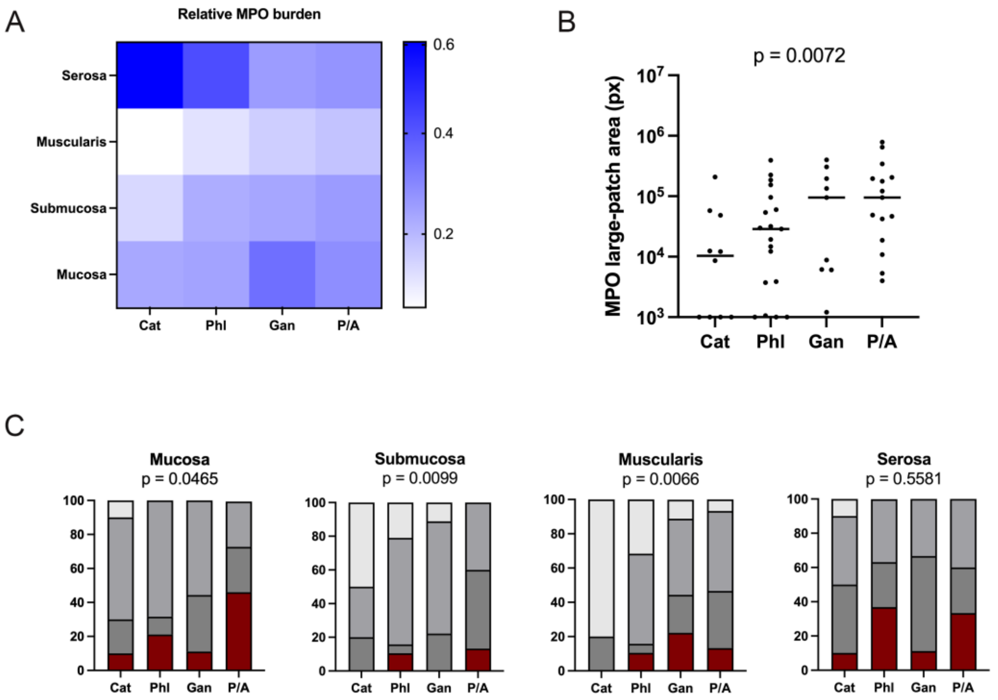

Supplementary Figure S5: MPO-positive large-patch analysis supports a less pronounced compartment-specific remodeling pattern

(A) Heatmap analysis reveals the relative distribution of MPO burden across appendiceal wall compartments. Color intensity represents the fraction of total MPO-positive burden within each histopathological category.

(B) Quantification of total MPO-positive large-patch area across catarrhal (Cat, n = 9), phlegmonous (Phl, n = 19), gangrenous (Gan, n = 9), and perforated/abscess appendicitis (P/A, n = 18).

(C) Layer-specific distribution of MPO-positive large-patch scores across appendiceal wall compartments (score 0–3). All displayed p-values indicate Cuzick trend test across ordered appendicitis stages.

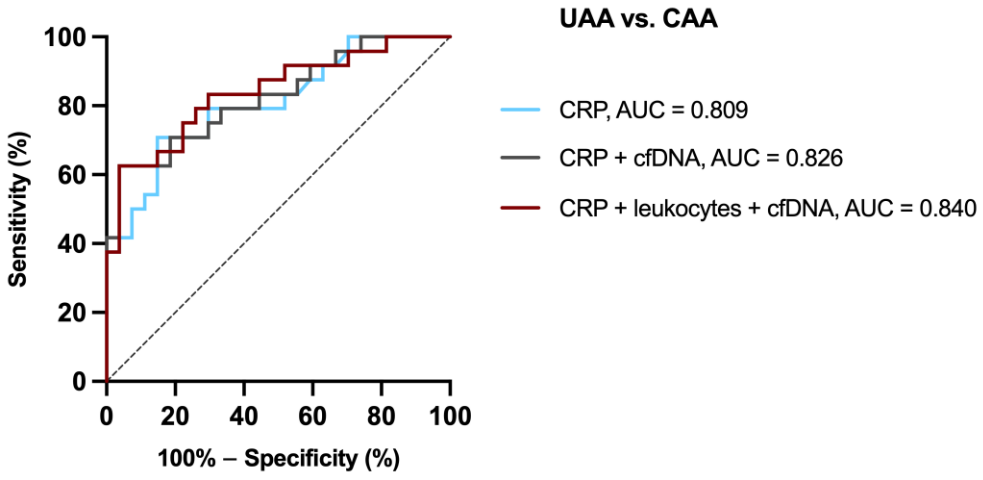

Supplementary Figure S6: Exploratory multivariable ROC models for discrimination of complicated appendicitis

ROC analysis for discrimination of uncomplicated appendicitis (UAA; catarrhal and phlegmonous appendicitis) from complicated

appendicitis (CAA; gangrenous and perforated/abscess appendicitis) using CRP, cfDNA, and multivariable combinations. The CRP + cfDNA model showed a numerically higher AUC than CRP alone but did not significantly improve discrimination (AUC 0.826 vs. 0.809; DeLong's test, $p = 0.2868$). The combined leukocyte + CRP + cfDNA model further increased the AUC numerically but was not significantly superior to CRP alone (AUC 0.840 vs. 0.809; DeLong's test, $p = 0.3274$).
